# Arabidopsis nitrate-induced aspartate oxidase gene expression is necessary to maintain metabolic balance under nitrogen nutrient fluctuation

**DOI:** 10.1038/s42003-022-03399-5

**Published:** 2022-05-09

**Authors:** Moriaki Saito, Mineko Konishi, Atsuko Miyagi, Yasuhito Sakuraba, Maki Kawai-Yamada, Shuichi Yanagisawa

**Affiliations:** 1 Agro-Biotechnology Research Center, Graduate School of Agricultural and Life Sciences, The University of Tokyo, Yayoi 1-1-1, Bunkyo-ku, Tokyo, 113-8657 Japan; 2 Graduate School of Science and Engineering, Saitama University, 255 Shimo-Okubo, Sakura-ku, Saitama, 338-8570 Japan; 3 Present Address: Department of Microbiology and Plant Pathology, University of California, Riverside, 900 University Ave., Riverside, CA USA; 4 Present Address: Faculty of Agriculture, Yamagata University, 1-23 Wakaba-machi, Tsuruoka-city, Yamagata 997-8555 Japan

**Keywords:** Plant signalling, Secondary metabolism

## Abstract

Nitrate is a nutrient signal that regulates growth and development through NLP transcription factors in plants. Here we identify the L-aspartate oxidase gene (*AO*) necessary for *de novo* NAD^+^ biosynthesis as an NLP target in Arabidopsis. We investigated the physiological significance of nitrate-induced *AO* expression by expressing *AO* under the control of the mutant *AO* promoter lacking the NLP-binding site in the *ao* mutant. Despite morphological changes and severe reductions in fresh weight, the loss of nitrate-induced *AO* expression resulted in minimum effects on NAD(H) and NADP(H) contents, suggesting compensation of decreased *de novo* NAD^+^ biosynthesis by reducing the growth rate. Furthermore, metabolite profiling and transcriptome analysis revealed that the loss of nitrate-induced *AO* expression causes pronounced impacts on contents of TCA cycle- and urea cycle-related metabolites, gene expression profile, and their modifications in response to changes in the nitrogen nutrient condition. These results suggest that proper maintenance of metabolic balance requires the coordinated regulation of multiple metabolic pathways by NLP-mediated nitrate signaling in plants.

## Abstract (web summary)

NLP transcription factors directly regulate aspartate oxidase gene expression connected to multiple metabolic pathways in Arabidopsis in response to changes in the nitrogen nutrient condition.

## Introduction

Nitrate, one of the main sources of nitrogen (N) for plants, plays an important role in the regulation of gene expression, metabolism, growth, and development^1,2^. Application of nitrate to N-starved plants induces various responses, collectively referred to as nitrate responses, which include the promotion of nitrate assimilation^3^, increase in lateral root growth^4^, changes in the shoot to root weight ratio^5^, and induction of the expression of N assimilation-related genes^2^. Furthermore, genome-wide studies revealed that nitrate induces the expression of not only N assimilation-related genes but also diverse genes involved in other metabolic pathways, such as the pentose phosphate pathway^6^. However, with the exception of N assimilation-related genes, the physiological significance of nitrate-induced expression of metabolic enzyme genes has not yet been elucidated.

NODULE INCEPTION-LIKE PROTEIN (NLP) transcription factors with a conserved N-terminal region and the RWP-RK DNA-binding domain are the primary transcription factors regulating nitrate-responsive gene expression in plants^7–10^. NLPs are post-translationally activated through nitrate-induced phosphorylation of a serine residue in the conserved N-terminal region^11^ and then accumulate in the nucleus^8^, where they interact with nitrate-responsive *cis*-elements (NREs)^10,12^ in target gene promoters to activate their expression. Of the nine NLPs in Arabidopsis, NLP7 plays a dominant role in regulating genes involved in nitrate utilization^13,14^, while NLP6, most closely related to NLP7, supports the role of NLP7^15^. Repression of the activity of the entire NLP family by chimeric repressor gene-silencing technology has been shown to diminish most of the nitrate-induced gene expression^7,10^. Furthermore, genome-wide analyses revealed the binding of NLP7 to the vicinity of numerous nitrate-inducible genes in the nucleus^8,16^.

NAD(H) and NADP(H), collectively referred to as pyridine nucleotides, are ubiquitous coenzymes required for a wide range of redox reactions in all living organisms^17^, because they deliver electrons via the reversible conversion of NAD^+^ and NADP^+^ to NADH and NADPH, respectively. NAD(H) is a redox carrier in the tricarboxylic acid (TCA) cycle and mitochondrial electron transport chain (ETC) in both plants and animals, whereas NADP(H) is a redox carrier in the pentose phosphate pathway and Photosystem I in plants. Thus, sufficient pools of NAD(H) and NADP(H) are necessary for maintaining the appropriate redox statuses in the cytosol, plastids, and mitochondria of plant cells^18^. In fact, partial defects of ETC have been shown to cause abnormalities in the redox status and reduction in N assimilation efficiency in *Nicotiana sylvestris*^19,20^. Defects in transporters comprising the malate valve, which delivers the reducing power of NADPH from the plastid to the cytosol in the form of malate, have also been shown to lower the efficiency of both carbon (C) and N metabolism, leading to the depletion of amino acids and accumulation of nitrate and organic acids^21^. Besides its primary role as a redox carrier, NAD^+^ also acts as a substrate in protein poly-ADP ribosylation and deacetylation, alternative RNA cap formation, and pathogen-induced cell death signaling^22–26^. Thus, NAD(H) content may also be associated with various physiological processes in a redox-independent manner^18,27^.

In the *de novo* NAD^+^ biosynthesis pathway in plants, aspartate is oxidized by aspartate oxidase (AO) to form iminoaspartate, which is converted to quinolinate by quinolinate synthase (QS)^28^. Then, quinolinate is converted into nicotinate mononucleotide (NaMN) by quinolinate phosphoribosyltransferase (QPT)^29^ and subsequently to nicotinate adenine dinucleotide by nicotinamide mononucleotide adenyltransferase (NMNAT)^30^, finally leading to the synthesis of NAD^+^ by NAD synthase (NADS)^31,32^ (Supplementary Fig. 1). The AO-catalyzed production of iminoaspartate is likely the rate-limiting step in *de novo* NAD^+^ biosynthesis because the NAD^+^ content per unit tissue fresh weight increased in Arabidopsis plants overexpressing *AO*^33^ and *QPT* overexpression enriched NAD^+^ content only when supplied with quinolinate^34^. Besides this *de novo* pathway, the salvage pathway regenerates NaMN via a three-step process composed of the degradation of NAD^+^ to form nicotinamide by poly (ADP-ribose) polymerase and sirtuins, deamidation of nicotinamide to nicotinate by nicotinamidase and conversion of nicotinate into NaMN by nicotinate phosphoribosyltransferase^35,36^ (Supplementary Fig. 1). Thus, AO, QS, and QPT are exclusively responsible for *de novo* NAD^+^ biogenesis, while NMNAT and NADS are involved in both *de novo* NAD^+^ biosynthesis and NAD^+^ salvage pathways (Supplementary Fig. 1). Because AO, QS, and QPT function in plastids, whereas NMNAT functions in the cytosol^37,38^, it is assumed that the rate of NAD^+^ biogenesis is regulated in the plastid, while NAD^+^ synthesized in the cytosol is transported to various subcellular organelles^39,40^. AO, QS, QPT, and NMNAT are encoded by single-copy genes in Arabidopsis^31^, and knockout mutations in *AO*, *QS*, *QPT*, and *NMNAT* genes cause embryonic lethality^37,41^. On the other hand, NADP^+^ and NADPH are generated by the phosphorylation of NAD^+^ and NADH, respectively, by NAD phosphotransferases (NADK1 and NADK2) and NADH phosphotransferase in Arabidopsis^42,43^. Analysis of *nadk2* knockout mutants and *NADK2* overexpression lines shows that the pool size of NADP^+^ and NADPH affects the regulation of amino acid metabolism and the Calvin cycle^44^.

In this study, we identified the *AO* gene as a direct target gene of the NLP family of transcriptional activators. Because the AO-catalyzed reaction is the key step in NAD^+^ biogenesis, we further explored the physiological significance of NLP-mediated nitrate induction of *AO* expression. The results suggest that nitrate-induced expression of *AO* plays a critical role in maintaining metabolic balance and growth in response to N nutrition, emphasizing the importance of the coordinated regulation of multiple metabolic pathways by nitrate signaling.

## Results

### NLP-mediated and nitrate-induced *AO* expression in Arabidopsis

**Fig. 1:**
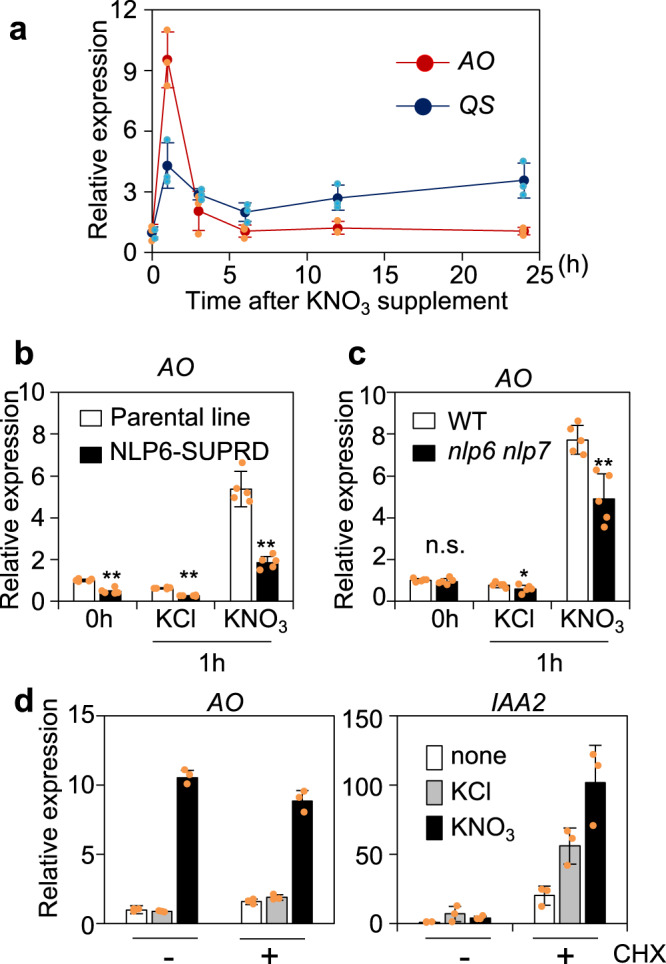
Nitrate-inducible expression of *AO*. **a** Time-course analysis of *AO* and *QS* expression after supplementation of KNO_3_ at the final concentration of 10 mM. Nitrate induction of expression of *AO* in NLP6-SURRD transgenic seedlings and the parental genotype (**b**), and in the wild-type (WT) and *nlp6 nlp7-1* double mutant seedlings (**c**). Asterisks indicate significant differences (Welch’s *t*-test; *P ≤ 0.05, **P ≤ 0.01). **d** Nitrate-induced expression of *AO* in plants treated with cycloheximide (CHX). In (**a**), 4-day-old WT seedlings were treated with 10 mM KNO_3_ for the indicated time period. In (**b**–**d**), seedlings were treated with 10 mM KNO_3_ or KCl (control) for 1 h. In (**d**), seedlings were treated with 100 µM CHX for 1 h before nitrate or control treatment. *IAA2* was used as a positive control gene for the CHX treatment. All seedlings were grown under continuous light. Data represent mean ± standard deviation [SD; *n* = 3 in (**a**, **d**) and *n* = 5 in (**b**, **c**)].

Our previous microarray data of NLP6-SUPRD transgenic lines, in which nitrate-induced expression of NLP target genes was diminished by expressing a dominant-negative chimeric repressor^7,10^, suggested that the expression of *AO* and *QS* is induced by nitrate-activated NLPs. Therefore, we performed a time-course analysis of *AO* and *QS* expression in Arabidopsis seedlings (Fig. 1a). Consistent with the microarray data, the expression of *AO* and *QS* was transiently induced by 10 mM KNO_3_ but not KCl (control), although the induction was more pronounced for *AO* than for *QS*. Furthermore, the nitrate-induced *AO* expression was reduced in both NLP6-SUPRD transgenic lines and the *nlp6 nlp7-1* double mutant (Figs. 1b, c). These results suggested that NLP6 and NLP7 directly or indirectly regulate nitrate-inducible *AO* expression, although a more evident reduction in the NLP6-SUPRD lines implied that other NLPs likely played redundant roles. The presence of cycloheximide, a protein synthesis inhibitor, did not affect the nitrate-induced expression of *AO* but induced the expression of *IAA2* (a treatment control)^45^ (Fig. 1d). Since NLPs are activated through nitrate-induced phosphorylation, *de novo* protein synthesis is unnecessary for increases in NLP activity. Therefore, this observation suggested that *AO* is directly regulated by NLPs. Consequently, *AO* was identified as a direct NLP target gene candidate.

### NLP7 binds to an NRE in the *AO* gene promoter to induce its expression

**Fig. 2:**
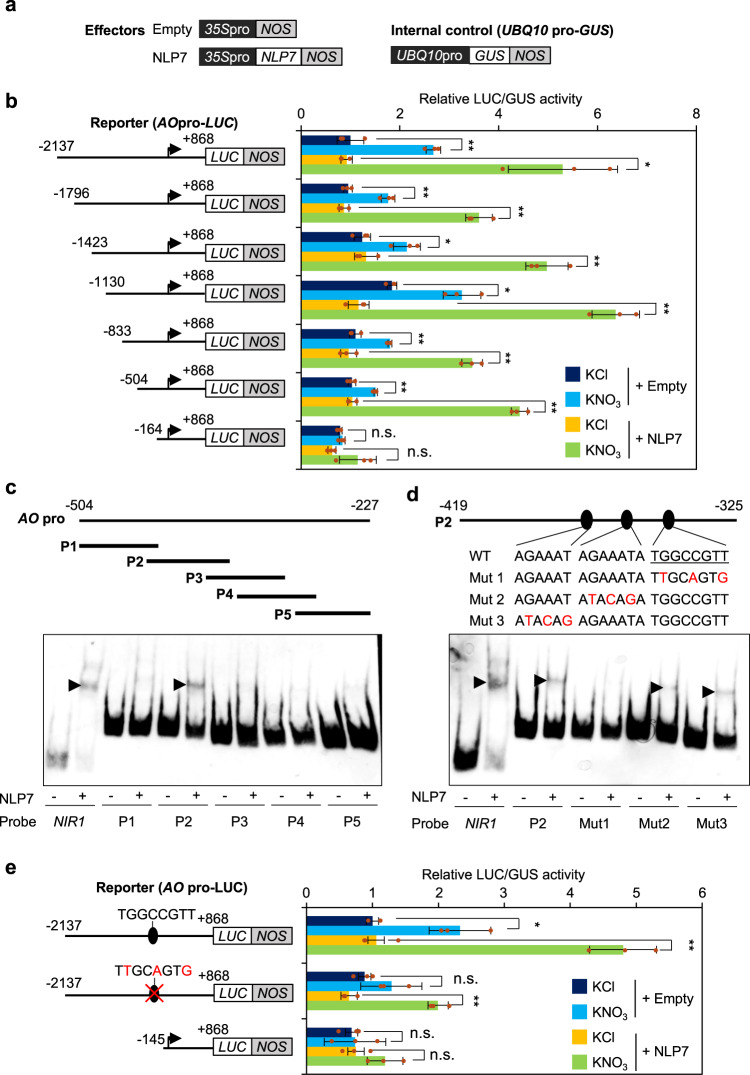
Nitrate-dependent activation of the *AO* promoter through the specific binding of NLP7. **a** Effector and internal control plasmids. The effector plasmid contains *NLP7* cDNA cloned between the modified *35* *S* promoter (*35S*pro) and the *NOS* terminator (*NOS*), while the empty vector contained no DNA insert. The plasmid carrying the GUS gene under the control of the *UBQ10* promoter (*UBQ10*pro) served as an internal control. **b** Co-transfection assay. Protoplasts from N-starved plants were co-transfected with a reporter plasmid (expressing the luciferase gene (*LUC*) under the control of a truncated fragment of the *AO* promoter), *NLP7* expression vector or the empty vector, and the internal control plasmid, and then incubated in the presence of 10 mM KNO_3_ or KCl (control). The transcription start sites are indicated by arrows. **c**, **d** Electrophoretic mobility shift assay (EMSA). DNA probes corresponding to different regions of the *AO* promoter (P1–P5) in (**c**) and the WT and mutant P2 probes in (**d**) were incubated in the presence (+) or absence (-) of recombinant NLP7. The mutant P2 probes (Mut1, Mut2, and Mut3) contained nucleotide substitutions (as indicated). A DNA fragment from the *NIR1* promoter was used as a positive control. Black arrowheads indicate retarded bands. **e** Activation of the *AO* promoter, depending on both the NLP7-binding site and NLP7. Reporter plasmids contained the WT or mutated *AO* promoter. The *AO* promoter truncated at -1,015 was used as a negative control. Nucleotide substitutions in the mutated *AO* promoter were the same as those in the Mut1 probe. In (**b**) and (**e**), data represent mean ± SD (*n* = 3), and relative LUC activity obtained from protoplasts co-transfected with the reporter plasmid (containing the full-length WT *AO* promoter) and the empty vector, and then incubated in the presence of 10 mM KCl, was set to 1. Asterisks indicate significant differences between seedlings treated with and without nitrate (Welch’s *t*-test; *P ≤ 0.05, **P ≤ 0.01). n.s. not significant.

To examine possible nitrate- and NLP-dependent activation of the *AO* promoter, co-transfection assays with protoplasts from N-starved plants were performed using the effector, reporter, and control plasmids (Figs. 2a, b). A 2137 bp-genomic sequence upstream of the transcription start site was employed as the *AO* promoter, considering that most functional cis-elements exist within 500 bp upstream of transcriptional start sites in the case of Arabidopsis genes^46^. In fact, this promoter succeeded in driving *AO* expression sufficient to complement the phenotype of the *ao* mutant, as described later. Reporter plasmids were constructed with the *AO* promoter and its derivatives truncated at approximately 300 bp intervals to facilitate the identification of NLP-binding sites. Protoplasts from N-starved plants co-transfected were incubated in the presence of 1 mM KNO_3_ or KCl (control). The results indicated that NLP7 activated the *AO* promoter truncated at position −504 (relative to the transcription start site) and other longer truncations of the *AO* promoter in the presence, but not in the absence, of 1 mM KNO_3_. However, the *AO* promoter truncated at −164 was activated neither by the nitrate treatment nor by NLP7 (Fig. 2b), suggesting that nitrate-activated NLP7 activated the *AO* promoter by binding to a site located between −504 and −164.

To precisely identify the NLP-binding site(s), an electrophoretic mobility shift assay (EMSA) was performed using recombinant NLP7 and a variety of DNA probes corresponding to the sequence between −504 and −164 of the *AO* promoter (Fig. 2c, d). Only probe P2 produced a shifted band, as did an NRE-containing fragment from the *NIR1* promoter^12^ (positive control) (Fig. 2c). Since probe P2 contained three DNA sequences resembling the consensus NLP-binding sequence, TG(A/G)C(C/T)CTT or its reverse complement AAG(G/A)G(T/C)CA^10^, EMSA was further performed with three mutant probes (Mut1–3) harboring nucleotide substitutions in any of the three putative NLP-binding sites (Fig. 2d). NLP7 bound to Mut2 and Mut3 probes, but not to the Mut1 probe, thus identifying the TGGCCGTT sequence (−354 to −347 bp in the *AO* promoter) as an NLP-binding site. The relevance of the identified NLP-binding site to the nitrate-inducible expression of *AO* was investigated by co-transfection assays (Fig. 2e). Unlike the wild-type *AO* promoter, the mutant *AO* promoter (harboring the same mutations as the Mut1 probe) was barely activated by nitrate when NLP7 was not overexpressed by co-transfection of the *NLP7* effector plasmid. Although overexpressed NLP7 caused small nitrate-dependent activation of the mutant *AO* promoter, the fold induction was much lower with the mutant *AO* promoter than with the wild-type *AO* promoter. Because the mutant *AO* promoter was not evidently activated in transgenic plants as described later, this small activation was probably due to a minor interaction between sequences somehow resembling the NLP-binding site and NLP7 overexpressed in the transient assay system. These results indicated that nitrate-activated NLP7 upregulates the *AO* promoter-dependent transcription by interacting with the TGGCCGTT sequence located between −354 and −347.

### Effects of the loss of nitrate-dependent *AO* expression on plant growth and morphology

**Fig. 3:**
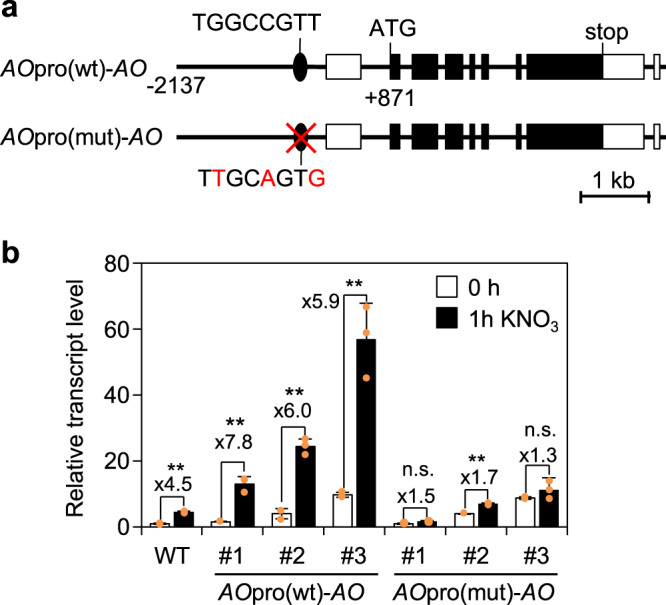
NLP-binding site-dependent induction of *AO* expression by nitrate *in planta*. **a** Schematic representation of *AO*pro(wt)-*AO* and *AO*pro(mut)-*AO* constructs introduced into the *ao* mutant. The construct contained a 6753 bp sequence of the *AO* locus (from 2,137 bp upstream of the transcription start site to 869 bp downstream of the stop codon). In the *AO*pro(mut)-*AO* construct, the NLP-binding sequence was mutated. White and black boxes indicate untranslated and translated regions, respectively. **b** RT-qPCR analysis of nitrate-induced *AO* expression in 4-day-old seedlings of the WT and three independent transgenic lines (#1, #2, and #3). The seedlings were treated with 10 mM KNO_3_ for 1 h. The expression level of *AO* in WT seedlings not exposed to the nitrate treatment was set to 1. Data represent mean ± SD (*n* = 3). Induction folds are indicated. Asterisks indicate significant differences between seedlings treated with and without nitrate (Welch’s *t*-test; *P ≤ 0.05, **P ≤ 0.01).

To determine the physiological significance of nitrate-induced *AO* expression, *AO* was expressed in the *ao* null mutant (*fin4-2*, SALK_013920)^47^ under the control of the wild-type *AO* promoter or mutant *AO* promoter with mutations on the identified NLP-binding site (Fig. 3a). Because the *ao* null mutant shows embryonic lethality^37^, plants heterozygous for the *ao* null allele were transformed. Then, T3 progenies homozygous for both the introduced *AO* gene and the *ao* allele were selected. The generated transgenic plants showed comparable or slightly higher levels of *AO* transcripts in the absence of nitrate. Since the transgenic lines whose T2 progenies showed a 3:1 segregation ratio of the selection marker gene were selected (see Methods), the slight differences in *AO* expression levels in transgenic lines could be due to multiple tandem insertions at a single gene locus or chromosomal position effect. On the other hand, nitrate-induced *AO* expression was clearly detected in all three independent transgenic lines generated using the wild-type *AO* promoter (*AO*pro(wt)-*AO* plants), while it was barely detectable in the three independent lines generated with the mutant *AO* promoter (*AO*pro(mut)-*AO* plants) (Fig. 3b). These results revealed that the identified NLP-binding site is a *cis*-acting element responsible for nitrate-inducible *AO* expression *in planta*.

**Fig. 4:**
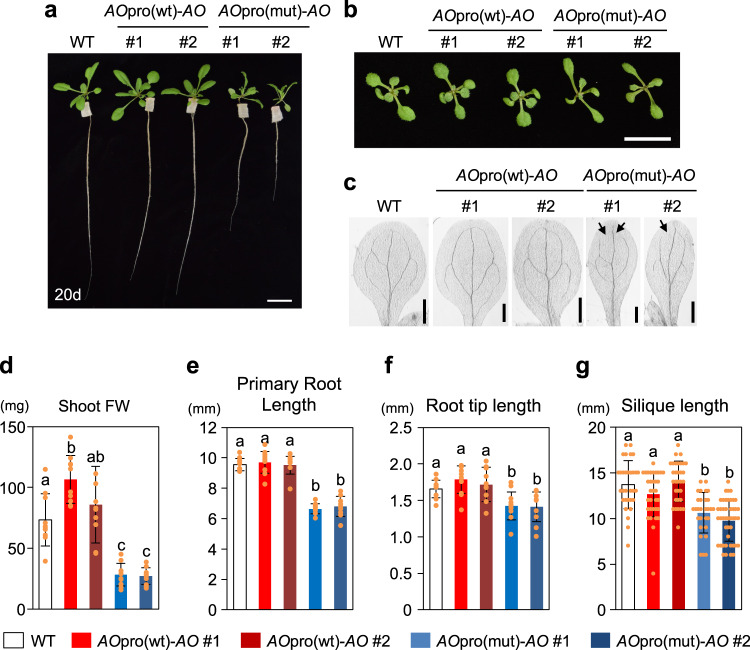
Phenotypes of *AO*pro(mut)-*AO* plants. **a** Images of shoot and root growth of seedlings grown hydroponically on 1/10MS medium for 20 days. Leaf shapes (**b**) and vascular patterns of cotyledons (**c**) of 10-day-old seedlings grown on agar plates. In (**c**), disconnected points of vascular loops are indicated by arrows. Scale bar = 2 cm (**a**), 1 cm (**b**), and 0.5 mm (**c**). **d** Fresh shoot weight of 20-day-old seedlings grown hydroponically on 1/10MS medium (*n* = 10). **e** Length of primary roots of 10-day-old seedlings grown on agar plates (*n* = 10–11). **f** Length of the meristematic region at the tip of the primary roots of 6-day-old seedlings grown on agar plates (*n* = 11–12). **g** Length of all siliques borne on the first flowering stem of plants grown on nutrient-supplemented soils under the long-day condition (*n* > 26). In (**d**–**g**), data represent mean ± SD, and different letters denote statistically significant differences (Tukey’s honestly significant difference [HSD] test; *P* < 0.05).

Phenotypic analysis of the generated transgenic lines revealed that *AO*pro(mut)-*AO* plants show growth defects and morphological abnormalities (Fig. 4, Supplementary Fig. 2). Unlike *AO*pro(wt)-*AO* plants, *AO*pro(mut)-*AO* plants showed decreases in shoot fresh weight and shorter primary root length (about 50% and 70%, respectively, compared to wild-type plants) (Fig. 4c, d). Furthermore, the *AO*pro(mut)-*AO* plants exhibited morphological abnormalities, such as narrower leaves with a disconnected vascular loop, shorter root tips, and shorter siliques (Fig. 4b, c, f, g). Defective lateral root development was also found in *AO*pro(mut)-*AO* seedlings grown on agar plates (Supplementary Fig. 2). The growth defects of *AO*pro(mut)-*AO* plants were restored at least partially by supplementation with nicotinamide or nicotinate, intermediate metabolites of the NAD^+^ salvage pathway (Supplementary Figs. 1 and 2). These results suggest that the loss of nitrate-induced expression of *AO* exerts pleiotropic effects on plant growth and development in Arabidopsis. We note that, consistent with these phenotypes, transgenic Arabidopsis plants expressing the β-glucuronidase (GUS) gene under the control of the *AO* promoter showed strong GUS staining in leaves, root-shoot junctions, main root-lateral root junctions, root tips, and pollen grains (Supplementary Fig. 3).

### Effects of the loss of nitrate-induced *AO* expression on NAD(H) and NADP(H) contents

**Fig. 5:**
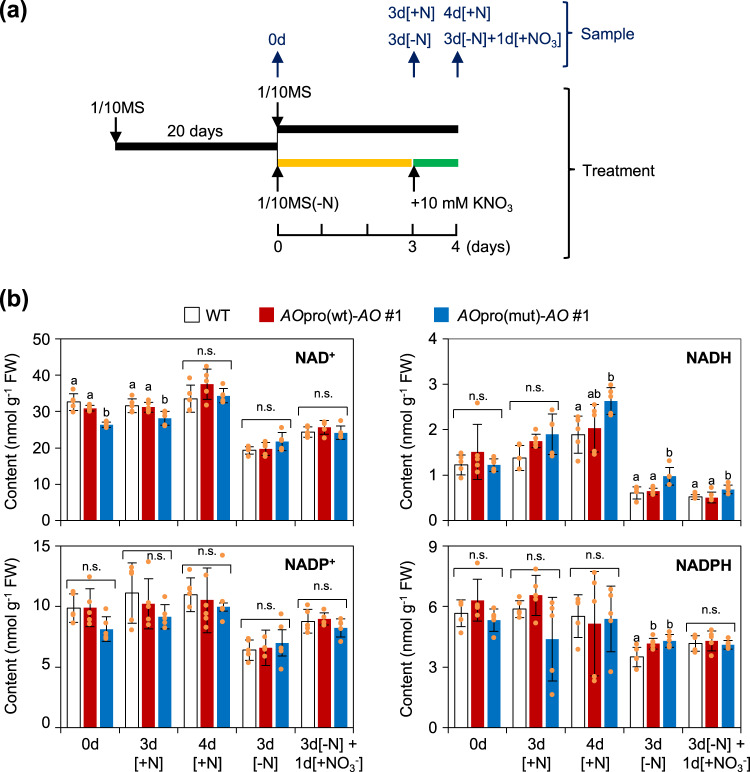
Dynamics of shoot NAD(H) and NADP(H) contents in response to changes in the N environment. **a** Experimental scheme for sampling time and N conditions. Sample 0d, plants grown hydroponically on 1/10MS medium for 20 d; sample 3d[-N], plants grown for 3 d on N-free 1/10MS medium after cultivation on 1/10MS medium for 20 d; sample 3d[-N3] + 1d[KNO_3_^-^], plants treated with 10 mM KNO_3_ at day 23. Control samples 3d[+N] and 4d[+N] represent plants grown hydroponically on 1/10MS medium for 20 days and then on fresh 1/10MS medium for 3 or 4 days, respectively. **b** NAD^+^, NADH, NADP^+^, and NADPH contents of the whole shoots of WT, *AO*pro(wt)-*AO*, and *AO*pro(mut)-*AO* plants sampled according to the scheme in (**a**). Data represent mean ± SD (*n* = 5). Different letters denote statistically significant differences (Tukey’s HSD test; *P* < 0.05).

The effects of the loss of the nitrate-dependent activation of *AO* expression on NAD(H) and NADP(H) contents were analyzed using the wild-type, *AO*pro(wt)-*AO*, and *AO*pro(mut)-*AO* plants, which were grown hydroponically in the N-free medium for 3 days to remove endogenous inorganic N and then in a 10 mM KNO_3_-containing medium for 1 day for *de novo* NAD synthesis requiring 5 enzymatic reactions (Supplementary Fig. 1), according to the scheme shown in Fig. 5a. We note that DNA microarray analysis in a later section revealed that before N starvation treatment, the expression *AO* level was about 2.75-fold lower on the log_2_ scale in *AO*pro(mut)-*AO* plants than *AO*pro(wt)-*AO* plants. The NAD^+^ content of *AO*pro(mut)-*AO* plants was lower (approximately 20%) than that of wild-type and *AO*pro(wt)-*AO* plants before N starvation treatment with a statistically significant difference (Fig. 5b). The N starvation treatment decreased the contents of NAD(H) and NADP(H), while KNO_3_ supply post-N starvation treatment increased these contents in plants of all genotypes used (Fig. 5b). However, the total contents of NAD(H) and NADP(H) in wild-type and *AO*pro(wt)-*AO* plants were significantly decreased by about 40% after the N starvation treatment and increased by about 20% after the KNO_3_ supply post-N starvation treatment (Welch’s *t*-test; *P* ≤ 0.01), whereas such changes in *AO*pro(mut)-*AO* plants were not statistically significant with only about 20% decrease and 10% increase, respectively. The total NAD(H) and NADP(H) contents after the KNO_3_ supply were still lower than the contents before the N starvation, likely because the *de novo* NAD biosynthesis was still in progress to restore the original level. These results suggest that the loss of nitrate-induced expression of *AO* attenuated the modifications of NAD(H) and NADP(H) contents in response to the N nutrient condition to a limited extent. These observations are in sharp contrast to the substantially reduced growth of *AO*pro(mut)-*AO* plants (Fig. 4). Therefore, these results meant that the negative effects of the loss of nitrate-induced *AO* expression are mostly compensated for by the decline in growth rate, consistent with the lethal phenotype of the *ao* mutant. Considering fresh weight per plant (Fig. 4), the total amount of NAD(H) and NADP(H) synthesized by *AO*pro(mut)-*AO* plants per individual was estimated to be less than half of that in the wild-type and *AO*pro(wt)-*AO* plants.

### Modified metabolite contents in *AO*pro(mut)-*AO* plants

**Fig. 6:**
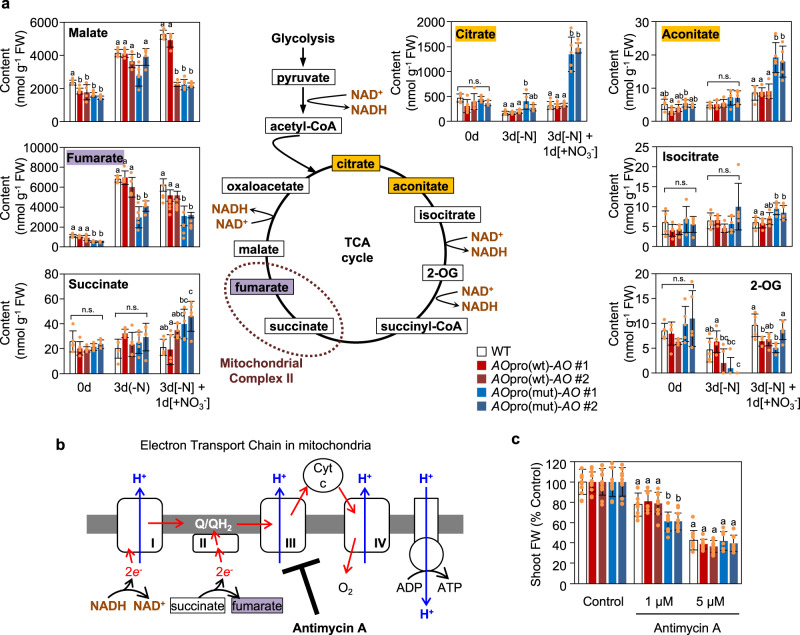
Abnormal accumulation of TCA cycle metabolites in *AO*pro(mut)-*AO* plants. **a** Contents of TCA cycle intermediates were measured in the shoots of WT, *AO*pro(wt)-*AO*, and *AO*pro(mut)-*AO* plants grown according to the scheme shown in Fig. 5a. Data represent mean ± SD (*n* = 5). **b** Schematic representation of the mitochondrial electron transport chain. Antimycin A inhibits the activity of Complex III. Q, ubiquinone; QH_2_, ubiquinol. **c** Effect of antimycin A on the shoot fresh weight. Seedlings were initially grown in the absence of antimycin A for 4 days and then in the presence of the indicated concentrations of antimycin A for 6 days (*n* = 7–12). Two independent lines (#1 and #2) of *AO*pro(wt)-*AO* and *AO*pro(mut)-*AO* plants were used. In (**a**, **c**), different letters denote statistically significant differences (Tukey’s HSD test; *P* < 0.05).

To identify the metabolic pathways that are strongly affected by the loss of the nitrate-induced *AO* expression, we performed a metabolomic analysis of the plants grown hydroponically according to the scheme shown in Fig. 5a. Measurement of metabolite contents in wild-type plants and two independent lines of each *AO*pro(wt)-*AO* and *AO*pro(mut)-*AO* genotypes revealed the contents of 68 metabolites including various organic acids and amino acids. As a result, the effect of selective abolishment of nitrate-induced *AO* expression was found to be more pronounced in the contents of TCA cycle-related organic acids but less prominent in the contents for amino acids and other groups of primary metabolites (Supplementary Data 1). For instance, before N starvation treatment, fumarate content in *AO*pro(mut)-*AO* plants was about half of those of wild-type and *AO*pro(wt)-*AO* plants (0d samples in Fig. 6, Supplementary Data 1). Consistent with the fact that fumarate is one of the most abundant metabolites in the TCA cycle, the total amount of metabolites in the TCA cycle was significantly lower in *AO*pro(mut)-*AO* plants (approximately 30% of that in wild-type plants) (Supplementary Data 1, Fig. 6a). Furthermore, metabolomic analysis clarified that the nutritional environment-induced changes in contents of TCA cycle-associated metabolites were similar in wild-type and *AO*pro(wt)-*AO* plants but distinct in *AO*pro(mut)-*AO* plants (Fig. 6a). Most evidently, in *AO*pro(mut)-*AO* plants, KNO_3_ supply post-N starvation treatment induced strong accumulation of citrate, another abundant metabolite in the TCA cycle, and succinate, which serve as substrates for the synthesis of 2-oxoglutarate (2-OG) necessary for N assimilation as carbon skeletons. Since the increases in citrate content in *AO*pro(mut)-*AO* plants (about 1000 nmol/g FW change) corresponded to about 15 % of the total organic acids in the TCA cycle, diminishing nitrate-inducible *AO* expression also exerted the pronounced effect on the composition of TCA metabolites, as shown by pie charts in Supplementary Fig. 4. These results revealed that nitrate-induced expression of *AO* is essential for proper maintenance of the TCA cycle in response to changes in the N nutrient environment.

The modified contents of TCA cycle-related organic acids in *AO*pro(mut)-*AO* plants may suggest attenuation of some enzymatic processes in the TCA cycle in *AO*pro(mut)-*AO* plants. Because fumarate is produced by mitochondrial complex II, a part of ETC, using NADH (Fig. 6b), we hypothesized that the low level of fumarate in *AO*pro(mut)-*AO* plants was caused by defects in the mitochondrial ETC. Consistent with this hypothesis, 1 µM antimycin A, an inhibitor of the ETC, inhibited the growth of *AO*pro(mut)-*AO* plants more effectively than that of wild-type and *AO*pro(wt)-*AO* plants (Fig. 6c). The *nlp6 nlp7-1* double mutant and the NLP6-SUPRD transgenic line also showed high sensitivity against 1 µM antimycin A (Supplementary Fig. 5), in line with the idea that NLP-mediated nitrate signaling impacts mitochondrial activity by modulating *AO* expression.

We note that, before N starvation treatment, the contents of arginine, proline, and several other urea cycle-related polyamines, such as putrescine and spermine, were different between *AO*pro(mut)-*AO* plants and the others, although the loss of nitrate-induced *AO* expression had little effect on the total amount of amino acids or the contents of most other amino acids (Supplementary Data 1, Fig. 7). Furthermore, these contents varied differently in WT and *AO*pro(wt)-*AO* plants and *AO*pro(mut)-*AO* plants. This result suggested another effect of the loss of nitrate-induced *AO* expression on primary metabolism.

**Fig. 7:**
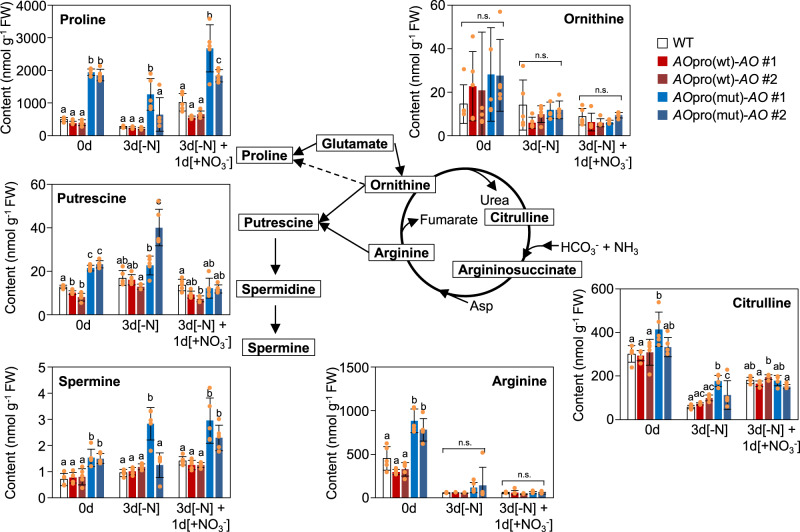
Abnormal accumulation of urea cycle-associated metabolites in *AO*pro(mut)-*AO* plants. Contents of urea cycle-associated metabolites were measured in the shoots of WT, *AO*pro(wt)-*AO*, and *Ao*pro(mut)-*AO* plants grown according to the scheme shown in Fig. 5a. Two independent lines (#1 and #2) of *AO*pro(wt)-*AO* and *AO*pro(mut)-*AO* plants were used. Data represent mean ± SD (*n* = 5). Different letters denote statistically significant differences (Tukey’s HSD test; *P* < 0.05).

Overall, compared to wild-type and *AO*pro(wt)-*AO* plants, *AO*pro(mut)-*AO* plants exhibited different accumulation of several TCA cycle- and urea cycle-related metabolites under any N nutrient conditions. These differences in metabolic balance, especially the differences under a normal steady growth condition before N starvation treatment, might be associated with the growth defect and morphological abnormality observed in Fig. 4 and Supplementary Fig. 2.

### Limited effects of loss of nitrate-induced *AO* expression on nitrate reduction and N assimilation

**Fig. 8:**
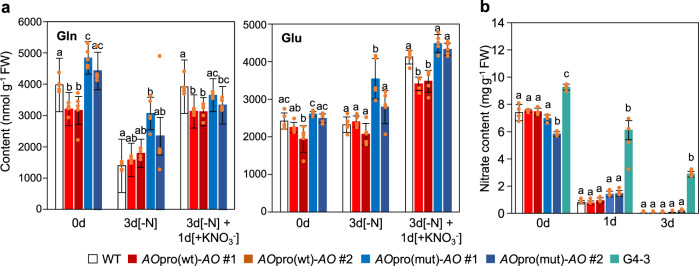
Limited effects of erasing nitrate-induced *AO* expression on glutamine and glutamate contents and decreases in nitrate content. **a** Glutamine and glutamate contents in the shoots of WT, *AO*pro(wt)-*AO*, and *AO*pro(mut)-*AO* plants grown according to the scheme shown in Fig. 5a. Two independent lines (#1 and #2) of *AO*pro(wt)-*AO* and *AO*pro(mut)-*AO* plants were used. Data represent mean ± SD (*n* = 5). **b** Decreases in the shoot nitrate content of wild-type, *AO*pro(wt)-*AO*, *AO*pro(mut)-*AO*, *nlp7* and G'4-3 seedlings during the N starvation treatment. Seedlings were first grown in the presence of KNO_3_ and NH_4_NO_3_ for 8 days and then grown in the absence of N sources for 0, 1, or 3 days. To grow G'4-3 (*nia1 nia2* double mutant) seedlings and other seedlings under the same nutrient condition, medium containing ammonium as N source was used in the pre-culture of all seedlings. Data represent mean ± SD (*n* = 4). In (**a**, **b**), different lowercase letters indicate significant differences among various genotypes (*P* < 0.05; Tukey’s HSD test).

Glutamine and glutamate contents were not notably different among wild-type, *AO*pro(wt)-*AO*, and *AO*pro(mut)-*AO* plants (Fig. 8a, Supplementary Data 1). Since glutamine and glutamate are the primary products of N assimilation and excellent metabolite markers for N assimilation activity^48^, this result suggested that the loss of nitrate-induced *AO* expression did not remarkably affect N assimilation and NADH-dependent reduction of nitrate. Therefore, we examined decreases in nitrate concentration upon N starvation in wild-type, *AO*pro(wt)-*AO*, and *AO*pro(mut)-*AO* plants and then found that these plants show a similar decrease in nitrate concentration upon N starvation (Fig. 8b). The observed declines in nitrate concentration were dependent on nitrate reductase (NR) because the decrease in nitrate concentration was very slow in seedlings of G'4-3, a *nia1 nia2* double mutant, which has only about 0.1% of the wild type NR activity in shoots and 5-10% of the wild type in roots^49,50^. Accordingly, these results suggest that the loss of nitrate-induced *AO* expression had no apparent effect on the nitrate reduction process as well as N assimilation.

### Effect of the loss of nitrate inducibility of *AO* expression on gene expression profiles

**Fig. 9:**
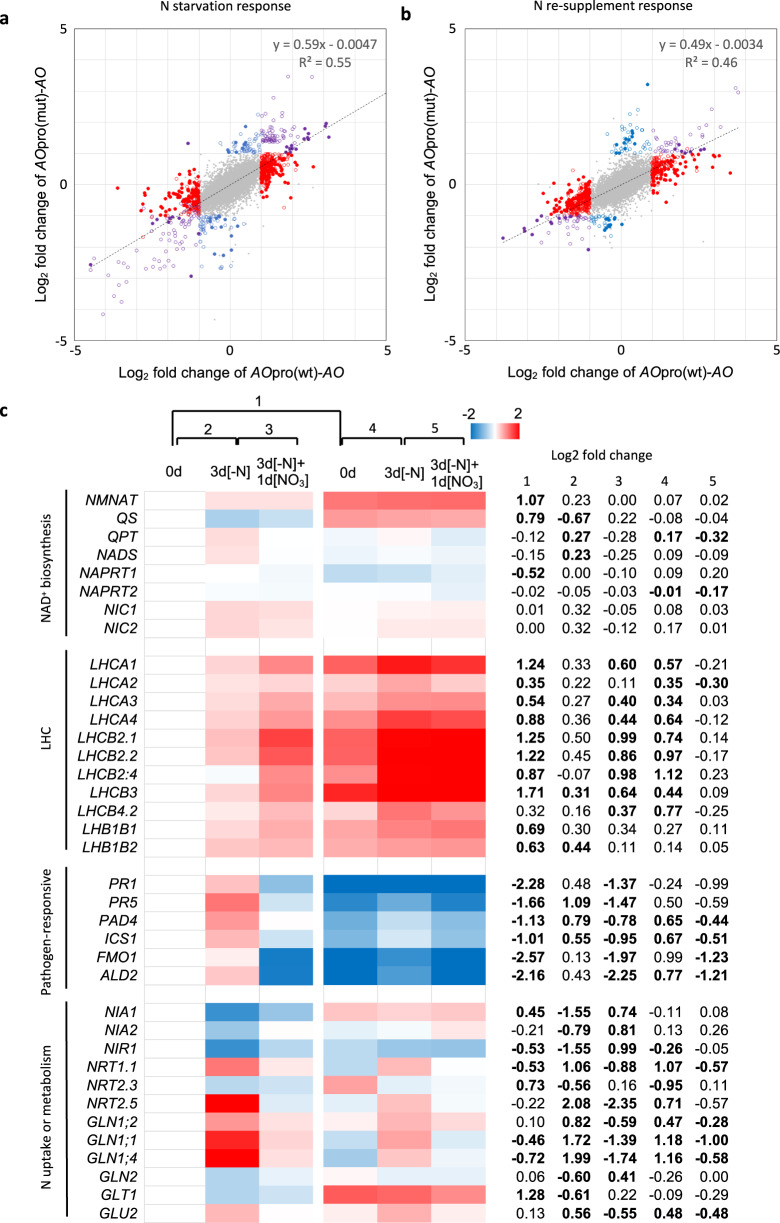
Effects of the loss of nitrate-induced *AO* expression on the transcriptome. Scatter plots of log_2_ fold changes in gene expression by N starvation treatment (0d vs. 3d[–N]) (**a**) or by nitrate resupply (3d[-N] vs. 3d[-N] + 1d[KNO_3_]) (**b**) in *AO*pro(wt)-*AO* and *AO*pro(mut)-*AO* plants. Gray dots indicate genes that did not significantly respond to N starvation treatment or KNO_3_ resupply, while open circles in red, blue, and purple indicate genes that significantly responded to N starvation treatment or KNO_3_ resupply in only *AO*pro(wt)-*AO*, only *AO*pro(mut)-*AO*, and both, respectively (log_2_ fold change ≥ |1|, adjusted *P*-value ≤ 0.05). Closed red, blue, and purple circles were applied when the adjusted P-value was less than 0.001. **c** Heatmap of typical genes whose expression levels were differentially modified in *AO*pro(wt)-*AO* vs. *AO*pro(mut)-*AO* plants during changes in the N conditions. Log_2_ fold changes (relative to the expression levels in sample 0d of *AO*pro(wt)-*AO* plants) are shown with a color scale. A chart on the right side of the heatmap shows log_2_ fold changes of expression levels in *AO*pro(mut)-*AO* to those in *AO*pro(wt)-*AO* plants before N starvation treatment (column 1), log_2_ fold changes caused by N starvation treatment (column 2) or nitrate resupply (column 3) in *AO*pro(wt)-*AO* plants, and log_2_ fold changes caused by N starvation treatment (column 4) or nitrate resupply (column 5) in *AO*pro(mut)-*AO* plants. Statistically significant differences are indicated in bold font (*P* < 0.05).

Based on the different dynamics of primary metabolite contents in wild-type, *AO*pro(wt)-*AO*, and *AO*pro(mut)-*AO* plants, we speculated that the loss of nitrate-induced *AO* expression might also cause unique modifications in the expression profiles in *AO*pro(mut)-*AO* plants through metabolite-mediated regulations. This possibility was examined by DNA microarray analysis of *AO*pro(wt)-*AO* and *AO*pro(mut)-*AO* plants grown according to the scheme shown in Fig. 5a. The results showed that before the N starvation treatment, 401 and 268 genes were upregulated and downregulated in *AO*pro(mut)-*AO* plants, respectively (*n* = 3, adjusted *P*-value ≤ 0.05, log_2_ fold change ≥ |1|) (Supplementary Data 2 and 3). Subsequent GO analysis revealed that these genes were enriched with photosynthesis, disease response, and senescence-related genes (Supplementary Data 4). Interestingly, both N starvation and KNO_3_ supply post-N starvation treatment modified the expression levels of a much smaller number of genes in *AO*pro(mut)-*AO* plants than in *AO*pro(wt)-*AO* plants (Supplementary Data 5–12). Furthermore, the slope of the linear regression lines representing the correlation between the changes in expression of each gene due to N starvation or KNO_3_ supply in *AO*pro(wt)-*AO* plants and *AO*pro(mut)-*AO* plants was less than 1 (0.59 and 0.49) (Fig. 9a, b). These results indicated that in *AO*pro(mut)-*AO* plants, the reactions to N starvation and KNO_3_ supply were attenuated in the gene expression profile, as were the modifications of NAD(H) and NADP(H) contents in response to the N nutritional change (Fig. 5b). GO analysis also supported this observation, showing that the GO terms enriched in *AO*pro(mut)-*AO* plants after N starvation or KNO_3_ supply, such as nitrate and salicylic acid response, were more highly enriched in *AO*pro(wt)-*AO* plants and also that the range of GO term repertoire was broader in *AO*pro(wt)-*AO* plants than in *AO*pro(mut)-*AO* plants (Supplementary Data 13-16).

The further analysis was performed using the Upset Shiny apps (https://gehlenborglab.shinyapps.io/upsetr/)^51^ to clarify overlaps among the lists of differentially expressed genes (Supplementary Fig. 6). As expected, many overlaps represented the genes similarly upregulated or downregulated by the same treatment in *AO*pro(wt)-*AO* and *AO*pro(mut)-*AO* plants. Most of the other overlaps were found between the lists of N starvation-responsive genes and genes whose expression levels were modulated in the opposite direction by KNO_3_ resupply. On the other hand, we also found that considerable overlaps between the list of differentially expressed genes in *AO*pro(mut)-*AO* plants before N starvation treatment and other gene lists barely existed, except for an overlap between the lists of the genes downregulated in *AO*pro(mut)-*AO* before N starvation treatment and the genes downregulated in *AO*pro(wt)-*AO* after KNO_3_ resupply, which was enriched with defense and senescence-related genes. Therefore, it is likely that the unique modifications in transcriptional profiles of *AO*pro(mut)-*AO* plants do not necessarily coincide with short-term nitrate signaling responses, but may also partly reflect the regulation associated with metabolites in nitrate signaling-regulated metabolic pathways.

Detailed analysis of differentially expressed genes in *AO*pro(wt)-*AO* and *AO*pro(mut)-*AO* plants revealed that a set of genes encoding light-harvesting complex (LHC) proteins were upregulated in *AO*pro(mut)-*AO* plants. In contrast, typical pathogen-responsive genes were downregulated (Fig. 9c, Supplementary Data 2 and 3). Furthermore, N starvation treatment and KNO_3_ supply resulted in relative increases in the expression of *LHC* genes only in *AO*pro(mut)-*AO* plants and only in *AO*pro(wt)-*AO* plants, respectively (column 2 vs. column 4 and column 3 vs. column 5 of the table inserted in Fig. 9c). Similarly, KNO_3_ supply post-N starvation treatment reduced the activation of pathogen-responsive genes more clearly in *AO*pro(wt)-*AO* plants. Furthermore, N nutrient condition-dependent modulations of the expression levels of several N metabolism-related genes were evident only in *AO*pro(wt)-*AO* plants (Fig. 9c). Collectively, the detailed analysis of differentially expressed genes also indicated that the modifications in gene expression in response to changes in the nutrient condition are generally blunted in *AO*pro(mut)-*AO* plants. We note an additional finding of slight but constitutive upregulation of *NMNAT* and *QS* genes in *AO*pro(mut)-*AO* plants (Fig. 9c), which may be a mechanism compensating for the relatively lower expression level of *AO* in *AO*pro(mut)-*AO* plants.

## Discussion

In the present study, the *AO* gene, encoding a key enzyme in *de novo* NAD^+^ biosynthesis, was identified as one of the genes directly regulated by nitrate-activated NLPs. Furthermore, by selectively abolishing the nitrate inducibility of *AO* expression *in planta*, we found that NLP-regulated nitrate-inducible *AO* expression is necessary to properly maintain metabolic balance and launch transcriptional adaptation of plants in response to changes in the N environment. Therefore, the present results provide direct evidence for the importance of coordinated regulation of multiple metabolic pathways by nitrate signaling in adaptation to the fluctuating N nutrient level, which is firstly demonstrated so far as we know. Since a number of metabolic enzyme genes are probably under the direct control of NLPs, other metabolic enzyme genes may also play critical roles in nitrate responses. Hence, this study suggests that future analysis of the nitrate-induced activation of metabolic enzyme genes in other metabolic pathways, as was done in this study, may reveal overlooked critical points to fully understand the role of nitrate as a plant growth regulator.

Phenotypic and metabolic analyses suggested that *AO*pro(mut)-*AO* plants relieved the negative effects of the abnormal regulation of *AO* expression by reducing their size rather than by decreasing NAD(H) and NADP(H) contents per unit tissue fresh weight. Since NAD(H) and NADP(H) are essential for growth, *AO*pro(mut)-*AO* plants probably had no choice but to restrict their growth in proportion to amounts of *de novo* synthesized NAD(H) and NADP(H) to survive. This conclusion means that there are minimum NAD(H) and NADP(H) contents to support plant growth, consistent with the lethal phenotype of *ao*, *qs*, *qpt*, and *nmnat* knockout mutants lacking the ability for *de novo* NAD^+^ biosynthesis^37,41^.

Metabolomic analysis revealed pronounced effects of the loss of the nitrate-induced *AO* expression on the TCA cycle rather than N assimilation (Supplementary Data 1, Fig. 6). Since an intermediate in the TCA cycle, 2-OG, is used as carbon skeletons required for N assimilation^52^, the nitrate responses may involve the enhancement of the TCA cycle as well as the N assimilation pathway. In fact, nitrate supply induced the expression of several genes encoding enzymes involved in the TCA cycle, leading to the accumulation of TCA cycle intermediates^53^. Therefore, a breakdown of nitrate-induced synchronized activation of enzymatic processes involved in 2-OG production and NAD^+^ biosynthesis probably caused remarked increases in the citrate content upon KNO_3_ supply (Fig. 6), emphasizing the importance of the nitrate-induced synchronized activation of enzymatic processes for the proper maintenance of the TCA cycle. Based on the low level of fumarate, low activity of mitochondrial ETC, and the irregular accumulation of intermediates and derivatives of the urea cycle in *AO*pro(mut)-*AO* plants (Fig. 7), we infer that nitrate-induced *AO* expression is tightly associated with the maintenance of the metabolic balance in mitochondria. On the other hand, selective abolishment of the nitrate-induced *AO* expression hardly affected the NADH-dependent reduction of nitrate in the cytosol, the first step in nitrate assimilation, as well as amino acid contents (Fig. 6). Therefore, we also speculate that the main reason for the induction of *AO* expression by nitrate may be maintaining the TCA cycle and other metabolic processes in mitochondria in response to nitrate supply.

At this stage, it is difficult to fully explain how the loss of nitrate-induced *AO* expression pronouncedly caused pleiotropic effects on metabolism and growth. However, considering that the most remarked effect of loss of nitrate-induced *AO* expression in the metabolite profile was the imbalance of metabolites related to the TCA cycle, which is central to primary metabolism, we assume that the loss of nitrate-induced *AO* expression first induces an imbalance in TCA cycle-related metabolites and then influences other metabolic pathways and growth *via* the imbalance. Although we cannot exclude the possibility that the loss of nitrate-induced *AO* expression also affects growth and development through other pathways, this hypothesis is consistent with the relatively weak variation in NAD(H) and NADP(H) contents and reduced responses to changes in the N nutrient condition in the gene expression profile in *AO*pro(mut)-*AO* plants. Furthermore, an imbalance in TCA cycle-related metabolites may cause indirect but widespread changes in gene expression profiles through metabolite-mediated regulations and other regulatory mechanisms. Further investigation of this hypothesis would be valuable in understanding how N nutrients promote plant growth.

## Methods

### Plant materials and growth conditions

*Arabidopsis thaliana* ecotype Columbia (Col) was used as the wild type. Seeds of the T-DNA insertion *ao* knockout mutant (*fin4-2*, SALK_013920)^46^ and G'4-3^49^ were obtained from the Arabidopsis Biological Resource Center (Columbus, OH, USA). The *nlp6 nlp7-1* double mutant, transgenic NLP6-SUPRD line, and its parental line were generated previously^7,54^. All plants were grown under continuous light (60–90 µE m^−2^ s^−1^) at 23 °C unless otherwise noted. To analyze nitrate-induced gene expression, seedlings were grown in N-free 1/10MS medium (N-free 1/10MS salts, 0.5 mM K_2_SO_4_, 0.1 g L^−1^ MES-KOH [pH 5.7]) supplemented with 0.5 mM ammonium succinate and 0.5 g L^−1^ sucrose for 4 days, and then treated with 10 mM KNO_3_ or KCl for 1 h in the presence or absence of 100 µM CHX. Around 100 seedlings were collected together as a biological replicate. We note that K_2_SO_4_ was added at a final concentration of 0.5 mM to prepare N-free 1/10MS medium because 1 mM K^+^ is sufficient to support the growth of Arabidopsis^55^. To conduct phenotypic analysis with agar plates, 4-day-old seedlings grown on agar plates (N-free 1/2MS salts, 10 mM KNO_3_, 1% sucrose, 0.8% agar) were further grown on fresh agar plates for 6–10 days. To examine the effects of antimycin A on the mitochondrial ETC, 4-day-old seedlings were transferred to agar plates with or without 1 or 5 µM antimycin A and grown for 6 days. To rescue the growth defects of *AO*pro(mut)-*AO* seedlings by supplementation with a metabolite, 4-day-old seedlings were transferred to agar plates containing 10 mM KNO_3_ in the presence or absence of 200 µM nicotinamide or 2 µM nicotinate and grown for 6 days. For phenotypic analysis, plants were also grown hydroponically on N-free 1/10MS medium supplemented with 10 mM KNO_3_ or on nutrient-containing peat moss (Jiffy-7, Sakata Seed Co., Kanagawa, Japan) under the long-day photoperiod (16 h light/8 h dark). To perform metabolic profiling and DNA microarray analysis, plants were grown hydroponically on 1/10MS medium for 20 days and then on N-free 1/10MS medium for 3 days. Subsequently, the plants were grown for 1 days on KNO_3_-supplemented medium (final concentration, 10 mM), while plants for reference samples were grown on 1/10MS medium for 3 or 4 days.

### Reverse transcription-quantitative PCR (RT-qPCR)

Total RNA for RT-qPCR was extracted from 4-day-old seedlings using the ISOSPIN Plant RNA Kit (NIPPON GENE Co., Tokyo, Japan). First-strand cDNA was synthesized using oligo(dT)_15_ primer (Invitrogen, CA, USA) and Superscript^TM^ II reverse transcriptase (Thermo Fisher, MA, USA). Quantitative PCR was performed on the StepOne Plus Real Time PCR System (Applied Biosystems, CA, USA) using the KAPA SYBR Fast qPCR Kit (KAPA Biosystems, MA, USA) and gene-specific primers (Supplementary Data 17). Expression levels of the genes of interest were normalized relative to that of *PEX4*^56^ or *UBQ10*.

### Plasmid construction

To construct reporter plasmids for the co-transfection assay, *AO* promoter fragments (from −2137 to +868, −1796 to +868, −1423 to +868, −1130 to +868, and −834 to +868, −504 to +868, and −164 to +868, relative to the transcription start site) were amplified by PCR using gene-specific primers. A mutated *AO* promoter fragment (−2137 to +868) carrying nucleotide substitutions within the NLP-binding site (−354 to −347 bp) was produced by overlap extension PCR. Then, the *35* *S* promoter and Ω enhancer region in the pJD301 vector were replaced by the obtained *AO* promoter fragments. Binary vectors for plant transformation were constructed by replacing the *35* *S* promoter and *GUS* gene in pCB302-HYG-35S-Ω-GUS^57,58^ with the wild-type or mutated *AO* promoter fragment (−2,137 to +868) and a PCR-amplified genomic DNA fragment (ranging from the translation initiation site at position +870 to 868 bp downstream of the stop codon), respectively. Another binary vector for histochemical analysis was generated by replacing only the *35S* promoter in pCB302-HYG-35S-Ω-GUS with the wild-type *AO* promoter (−2137 to +868). All primers used for plasmid construction are listed in Supplementary Data 17. All PCR products used for plasmid construction were verified by DNA sequencing.

### Co-transfection assay

Mesophyll protoplasts were prepared from wild-type Arabidopsis treated with N-starvation by the Tape-Arabidopsis Sandwich method^59^. Co-transfection with the effector, reporter, and control plasmids was performed by the DNA-PEG–calcium transfection method^60^. Effector plasmids used were p35SC4PPDK-none-MYC and p35SC4PPDK-NLP7-MYC^7^, and pUBQ10-GUS was used as a control plasmid^7^. Measurement of luciferase (LUC) and GUS activity was performed using the Luciferase Assay System (Promega Co., WI, USA) and 4-methylumbelliferyl-β-D-glucuronide, respectively^54^.

### EMSA

The His-tagged RWP-RK DNA-binding domain of NLP7 (recombinant NLP7 protein) was prepared by affinity chromatography using Ni-NTA resin^7^. Biotin-labeled DNA probes were generated by PCR using specific primers. The mutated DNA probes (Mut1–3), harboring point mutations in the putative NLP-binding site, were prepared by overlap extension PCR or by PCR with a primer harboring the Mut1 sequence. All primers used are listed in Supplementary Data 17. The recombinant NLP7 was incubated together with probe DNAs in the binding buffer (0.5 µg poly [d(I-C)], 20 mM HEPES-KOH (pH 7.6), 3 mM MgCl_2_, 2 mM dithiothreitol, 0.5 mM EDTA, 30 mM KCl, 30 mM NaCl, and 10% glycerol) at room temperature. After gel electrophoresis of the reaction mixtures, the probe DNAs were transferred from gels onto nylon membranes and detected using streptavidin-conjugated horseradish peroxidase^7^.

### Generation of transgenic Arabidopsis plants

Plants heterozygous for the *ao* null allele were transformed by the floral dip method^61^ using *Agrobacterium tumefaciens* strain GV3101 (pMP90)^62^ harboring a binary vector. T2 lines showing a 3:1 (hygromycin B resistant: sensitive) segregation ratio were selected. Then, T3 progenies homozygous for both the introduced gene and the *ao* null allele were selected by PCR-based genotyping using gene-specific primers (Supplementary Data 17). T3 or T4 progenies were used for analyses.

### Analysis of vascular patterns

Cotyledons were fixed in a mixture of ethanol and acetic acid (9:1), hydrated using a graded series of ethanol, and then mounted with the clearing solution^63^. Vascular patterns were analyzed using a stereomicroscope (MZ16F; Leica Microsystems, Germany).

### Histochemical GUS staining

Wild-type Arabidopsis plants were transformed with a binary vector harboring the *GUS* gene under the control of the wild-type *AO* promoter. Homozygous T3 progenies were selected and used for GUS staining. Briefly, 10-day-old seedlings grown on 1/2MS agar plates and floral buds and stamens collected from 40-day-old plants grown in the soil were fixed in acetone at −20 °C and washed with 0.1 M sodium phosphate buffer (pH 7.4). For GUS staining, samples were incubated in X-Glc solution (0.5 g L^−1^ 5-bromo-4-chloro-3-indolyl-*β*-D-glucuronide cyclohexylammonium salt, 0.5 mM potassium ferricyanide, 0.5 mM potassium ferrocyanide, and 0.1 M sodium phosphate, pH 7.4) at 37 °C^12^.

### Measurement of metabolite contents

Aerial tissues of each plant grown according to the scheme shown in Fig. 5a were harvested and immediately frozen in liquid N_2_. Two shoots (*AO*pro(wt)-*AO* plants) or three-five shoots (*AO*pro(mut)-*AO* plants) were pooled to form one independent biological replicate. NAD^+^, NADH, NADP^+^, and NADPH were extracted from frozen tissues and quantified by a plate reader method of Queval and Noctor^64^. Metabolite analysis was performed using the capillary electrophoresis coupled with triple quadrupole mass spectrometry (CE-LC/MS/MS) system (CE, 7100; MS, G6420A; Agilent Technologies, CA, USA)^65^.

### Measurement of nitrate content

Nitrate content was measured using the shoots of seedlings that were grown on N-free 1/2MS agar plates supplemented with 3.3 mM KNO_3_ and 3.3 mM NH_4_NO_3_ for 8 days and then grown on N-free 1/2MS agar plates for 0, 1, or 3 days. The Nitrate Test Kit (NECi Superior Enzymes, MI, USA) was used according to the manufacturer’s instructions.

### DNA microarray analysis

Total RNA was extracted from the whole shoots of *AO*(wt)-*AO* (line #1) and *AO*(mut)-*AO* (line #1) plants grown according to the scheme shown in Fig. 5a, using the ISOSPIN Plant RNA Kit. Cyanine-3-labeled cRNA was prepared with the Low Input Quick Amp Labeling Kit (One-Color; Agilent Technologies). We used the shoot of two or three seedlings to make a biological replicate, and biological triplicates were used for DNA microarray analysis. The labeled cRNA was hybridized to the Arabidopsis (V4) Oligo Microarray (Agilent Technologies), and the DNA Microarray Scanner (G2565BA; Agilent Technologies) was used to scan the arrays. The scanned images were analyzed with Feature Extraction (v9.1) (Agilent Technologies). Data analysis was performed using a statistical R language^66,67^. The LIMMA package was used for linear modeling to acquire empirical Bayes statistics^66^. Differentially expressed genes with an adjusted *P*-value ≤ 0.05 and absolute log_2_ fold change ≥ |1| were extracted for each comparison. Effects of genotype-environment interactions were examined by creating a contrast from two contrasts of each genotype before and after the treatments (*P*-value ≤ 0.001). Visualizing the intersections across differentially expressed gene lists was done with Upset Shiny apps (https://gehlenborglab.shinyapps.io/upsetr/)^51^. Gene ontology term enrichment analysis was done with the PANTHER classification system (http://pantherdb.org/). Microarray data were deposited in the Gene Expression Omnibus (GEO) repository of the National Center for Biotechnology Information (NCBI) database under the accession number GSE136542 on August 28, 2019.

### Statistics and reproductivity

All data shown in graphs are presented as the mean with S.D. from more than three biological replicates. Statistical significance was tested by Welch’s *t*-test (*P ≤ 0.05, **P ≤ 0.01) or Tukey’s honestly significant difference [HSD] test (Different lowercase letters indicate significant differences, *P* < 0.05).

### Reporting summary

Further information on research design is available in the Nature Research Reporting Summary linked to this article.

## Supplementary information

Supplementary information

Description of Additional Supplementary Files

Supplementary Data 1

Supplementary Data 2

Supplementary Data 3

Supplementary Data 4

Supplementary Data 5

Supplementary Data 6

Supplementary Data 7

Supplementary Data 8

Supplementary Data 9

Supplementary Data 10

Supplementary Data 11

Supplementary Data 12

Supplementary Data 13

Supplementary Data 14

Supplementary Data 15

Supplementary Data 16

Supplementary Data 17

Reporting Summary

## Data Availability

All materials are available from the corresponding author upon reasonable request.

## References

[1] Crawford NM (1995). Nitrate: Nutrient and signal for plant growth. Plant Cell.

[2] Wang Y-Y, Cheng Y-H, Chen K-E, Tsay Y-F (2018). Nitrate transport, signaling, and use efficiency. Annu. Rev. Plant Biol..

[3] Campbell WH (1988). Nitrate reductase and its role in nitrate assimilation in plants. Physiol. Plant..

[4] Zhang H, Jennings A, Barlow PW, Forde BG (1999). Dual pathways for regulation of root branching by nitrate. Proc. Natl Acad. Sci. USA.

[5] Scheible W-R, Lauerer M, Schulze E-D, Caboche M, Stitt M (1997). Accumulation of nitrate in the shoot acts as a signal to regulate shoot-root allocation in tobacco. Plant J..

[6] Wang R, Okamoto M, Xing X, Crawford NM (2003). Microarray analysis of the nitrate response in Arabidopsis roots and shoots reveals over 1,000 rapidly responding genes and new linkages to glucose, trehalose-6-phosphate, iron, and sulfate metabolism. Plant Physiol..

[7] Konishi M, Yanagisawa S (2013). Arabidopsis NIN-like transcription factors have a central role in nitrate signalling. Nat. Commun..

[8] Marchive C (2013). Nuclear retention of the transcription factor NLP7 orchestrates the early response to nitrate in plants. Nat. Commun..

[9] Krapp A (2014). Nitrate transport and signalling in Arabidopsis. J. Exp. Bot..

[10] Konishi M, Yanagisawa S (2014). Emergence of a new step towards understanding the molecular mechanisms underlying nitrate-regulated gene expression. J. Exp. Bot..

[11] Liu K (2017). Discovery of nitrate–CPK–NLP signalling in central nutrient–growth networks. Nature.

[12] Konishi M, Yanagisawa S (2010). Identification of a nitrate-responsive *cis*-element in the Arabidopsis *NIR1* promoter defines the presence of multiple *cis*-regulatory elements for nitrogen response. Plant J..

[13] Castaings L (2009). The nodule inception-like protein 7 modulates nitrate sensing and metabolism in Arabidopsis. Plant J..

[14] Konishi M, Okitsu T, Yanagisawa S (2021). Nitrate-responsive NIN-like protein (NLP) transcription factors perform unique and redundant roles in Arabidopsis. J. Exp. Bot..

[15] Guan P (2017). Interacting TCP and NLP transcription factors control plant responses to nitrate availability. Proc. Natl Acad. Sci. USA.

[16] Alvarez JM (2020). Transient genome-wide interactions of the master transcription factor NLP7 initiate a rapid nitrogen-response cascade. Nat. Commun..

[17] Berger F, Ramírez-Hernández MH, Ziegler M (2004). The new life of a centenarian: signalling functions of NAD(P). Trends Biochem. Sci..

[18] Hashida S, Takahashi H, Uchimiya H (2009). The role of NAD biosynthesis in plant development and stress responses. Ann. Bot..

[19] Hager J (2010). Conditional modulation of NAD levels and metabolite profiles in *Nicotiana sylvestris* by mitochondrial electron transport and carbon/nitrogen supply. Planta.

[20] Dutilleul C (2005). Mitochondria-driven changes in leaf NAD status exert a crucial influence on the control of nitrate assimilation and the integration of carbon and nitrogen metabolism. Plant Physiol..

[21] Kinoshita H (2011). The chloroplastic 2-oxoglutarate/malate transporter has dual function as the malate valve and in carbon/nitrogen metabolism. Plant J..

[22] Briggs AG, Bent AF (2011). Poly(ADP-ribosyl)ation in plants. Trends Plant Sci..

[23] Houtkooper RH, Pirinen E, Auwerx J (2012). Sirtuins as regulators of metabolism and healthspan. Nat. Rev. Mol. Cell Biol..

[24] Zhang H, Zhao Y, Zhou D-X (2017). Rice NAD^+^-dependent histone deacetylase OsSRT1 represses glycolysis and regulates the moonlighting function of GAPDH as a transcriptional activator of glycolytic genes. Nucleic Acids Res.

[25] Zhang H (2019). NAD tagSeq reveals that NAD^+^-capped RNAs are mostly produced from a large number of protein-coding genes in *Arabidopsis*. Proc. Natl Acad. Sci. USA.

[26] Wan L (2019). TIR domains of plant immune receptors are NAD^+^-cleaving enzymes that promote cell death. Science.

[27] Pétriacq P, de Bont L, Tcherkez G, Gakière B (2013). NAD: not just a pawn on the board of plant-pathogen interactions. Plant Signal. Behav..

[28] Nasu S, Wicks FD, Gholson RK (1982). L-Aspartate oxidase, a newly discovered enzyme of *Escherichia coli*, is the B protein of quinolinate synthetase. J. Biol. Chem..

[29] Bhatia R, Calvo KC (1996). The sequencing, expression, purification, and steady-state kinetic analysis of quinolinate phosphoribosyl transferase from *Escherichia coli*. Arch. Biochem. Biophys..

[30] Mehl RA, Kinsland C, Begley TP (2000). Identification of the *Escherichia coli* nicotinic acid mononucleotide adenylyltransferase gene. J. Bacteriol..

[31] Katoh A, Hashimoto T (2004). Molecular biology of pyridine nucleotide and nicotine biosynthesis. Front. Biosci..

[32] Willison JC, Tissot G (1994). The *Escherichia coli efg* gene and the *Rhodobacter capsulatus adgA* gene code for NH_3_-dependent NAD synthetase. J. Bacteriol..

[33] Hao J, Pétriacq P, de Bont L, Hodges M, Gakière B (2018). Characterization of L-aspartate oxidase from Arabidopsis thaliana. Plant Sci..

[34] Pétriacq P (2012). Inducible NAD overproduction in Arabidopsis alters metabolic pools and gene expression correlated with increased salicylate content and resistance to *Pst-AvrRpm1*. Plant J..

[35] Frothingha R, Meeker-O’Connell WA, Talbot EAS, George JW, Kreuzer KN (1996). Identification, cloning, and expression of the *Escherichia coli* pyrazinamidase and nicotinamidase gene, *pncA*. Antimicrob. Agents Chemother..

[36] Wubbolts MG (1990). Variation of cofactor levels in *Escherichia coli*: Sequence analysis and expression of the *pncB* gene encoding nicotinic acid phosphoribosyltransferase. J. Biol. Chem..

[37] Katoh A, Uenohara K, Akita M, Hashimoto T (2006). Early steps in the biosynthesis of NAD in Arabidopsis start with aspartate and occur in the plastid. Plant Physiol..

[38] Hashida S (2010). Nicotinate/nicotinamide mononucleotide adenyltransferase-mediated regulation of NAD biosynthesis protects guard cells from reactive oxygen species in ABA-mediated stomatal movement in *Arabidopsis*. J. Exp. Bot..

[39] Palmieri F (2009). Molecular identification and functional characterization of *Arabidopsis thaliana* mitochondrial and chloroplastic NAD^+^ carrier proteins. J. Biol. Chem..

[40] van Roermund (2016). The peroxisomal NAD carrier from Arabidopsis imports NAD in exchange with AMP. Plant Physiol..

[41] Hashida S, Takahashi H, Kawai-Yamada M, Uchimiya H (2007). *Arabidopsis thaliana* nicotinate/nicotinamide mononucleotide adenyltransferase (AtNMNAT) is required for pollen tube growth. Plant J..

[42] Turner WL, Waller JC, Vanderbeld B, Snedden WA (2004). Cloning and characterization of two NAD kinases from Arabidopsis. Identification of a calmodulin binding isoform. Plant Physiol..

[43] Turner WL, Waller JC, Snedden WA (2005). Identification, molecular cloning and functional characterization of a novel NADH kinase from *Arabidopsis thaliana* (thale cress). Biochem. J..

[44] Takahashi H (2009). Pleiotropic modulation of carbon and nitrogen metabolism in Arabidopsis plants overexpressing the NAD kinase2 gene. Plant Physiol..

[45] Abel S, Oeller PW, Theologist A (1994). Early auxin-induced genes encode short-lived nuclear proteins. Biochemistry.

[46] Korkuć P, Schippers HMJ, Walther D (2014). Characterization and identification of cis-regulatory elements in arabidopsis based on single-nucleotide polymorphism information. Plant Physiol..

[47] Macho PA, Boutrot F, Rathjen PJ, Zipfel C (2012). Aspartate oxidase plays an important role in arabidopsis stomatal immunity. Plant Physiol..

[48] Lea PJ, Miflin BJ (2003). Glutamate synthase and the synthesis of glutamate in plants. Plant Physiol. Biochem..

[49] Wilkinson JQ, Crawford NM (1993). Identification and characterization of a chlorate-resistant mutant of *Arabidopsis thaliana* with mutations in both nitrate reductase structural genes *NIA1* and *NIA2*. Mol. Gen. Genet..

[50] Lejay L (1999). Molecular and functional regulation of two NO_3_^-^ uptake systems by N- and C-status of Arabidopsis plants. Plant J..

[51] Lex A, Gehlenborg N, Strobelt H, Vuillemot R, Pfister H (2014). UpSet: Visualization of intersecting sets. IEEE Trans. Vis. Comput. Graph..

[52] Nunes-Nesi A, Fernie AR, Stitt M (2010). Metabolic and signaling aspects underpinning the regulation of plant carbon nitrogen interactions. Mol. Plant.

[53] Scheible W-R (1997). Nitrate acts as a signal to induce organic acid metabolism and repress starch metabolism in tobacco. Plant Cell.

[54] Maeda Y (2018). A NIGT1-centred transcriptional cascade regulates nitrate signalling and incorporates phosphorus starvation signals in *Arabidopsis*. Nat. Commun..

[55] Zhao S, Zhang M-L, Ma T-L, Wang Y (2016). Phosphorylation of ARF2 relieves its repression of transcription of the K+ transporter gene *HAK5* in response to low potassium stress. Plant Cell.

[56] Czechowski T, Stitt M, Altmann T, Udvardi MK, Scheible WR (2005). Genome-wide identification and testing of superior reference genes for transcript normalization in Arabidopsis. Plant Physiol..

[57] Kato Y, Konishi M, Shigyo M, Yoneyama T, Yanagisawa S (2010). Characterization of plant eukaryotic translation initiation factor 6 (eIF6) genes: The essential role in embryogenesis and their differential expression in *Arabidopsis* and rice. Biochem. Biophys. Res. Commun..

[58] Konishi M, Yanagisawa S (2007). Sequential activation of two Dof transcription factor gene promoters during vascular development in *Arabidopsis thaliana*. Plant Physiol. Biochem..

[59] Wu F-H (2009). Tape-*Arabidopsis* Sandwich—a simpler *Arabidopsis* protoplast isolation method. Plant Methods.

[60] Yoo S-D, Cho Y-H, Sheen J (2007). Arabidopsis mesophyll protoplasts: a versatile cell system for transient gene expression analysis. Nat. Protoc..

[61] Clough SJ, Bent AF (1998). Floral dip: a simplified method for *Agrobacterium*-mediated transformation of *Arabidopsis thaliana*. Plant J..

[62] Koncz C, Schell J (1986). The promoter of T_L_-DNA gene *5* controls the tissue-specific expression of chimaeric genes carried by a novel type of Agrobacterium binary vector. MGG Mol. Gen. Genet..

[63] Konishi M, Sugiyama M (2003). Genetic analysis of adventitious root formation with a novel series of temperature-sensitive mutants of *Arabidopsis thaliana*. Development.

[64] Queval G, Noctor G (2007). A plate reader method for the measurement of NAD, NADP, glutathione, and ascorbate in tissue extracts: Application to redox profiling during Arabidopsis rosette development. Anal. Biochem..

[65] Miyagi A (2020). Metabolome analysis of rice leaves to obtain low-oxalate strain from ion beam-mutagenised population. Metabolomics.

[66] Ihaka R, Gentleman R (1996). R: A language for data analysis and graphics. J. Comput. Graph. Stat..

[67] Ritchie ME (2015). Limma powers differential expression analyses for RNA-sequencing and microarray studies. Nucleic Acids Res.

