# Supplementary material for: Arabidopsis nitrate-induced aspartate oxidase gene expression is necessary to maintain metabolic balance under nitrogen nutrient fluctuation
Source: Commun Biol. 2022 May 9;5:432. doi: 10.1038/s42003-022-03399-5 (PMC9085827; doi:10.1038/s42003-022-03399-5)
Supplement: Supplementary file 1 — Supplementary information [file 42003_2022_3399_MOESM1_ESM.pdf]

Supplementary information

Arabidopsis nitrate-induced aspartate oxidase gene expression necessary to maintain metabolic balance under nitrogen nutrient fluctuation

Moriaki Saito[1,†], Mineko Konishi[1], Atsuko Miyagi[2,‡], Yasuhito Sakuraba[1], Maki Kawai-Yamada[2], & Shuichi Yanagisawa[1,*]

[1]Agro-Biotechnology Research Center, Graduate School of Agricultural and Life Sciences, The University of Tokyo, Yayoi 1-1-1, Bunkyo-ku, Tokyo 113-8657, Japan
[2]Graduate School of Science and Engineering, Saitama University, 255 Shimo-Okubo, Sakura-ku, Saitama, 338-8570, Japan

[†]Current address: Department of Microbiology and Plant Pathology, University of California, Riverside, 900 University Ave., Riverside, CA, USA
[‡]Current address: Faculty of Agriculture, Yamagata University, 1-23 Wakaba-machi, Tsuruoka-city, Yamagata 997-8555, Japan

*Corresponding author:
Shuichi Yanagisawa
Tel: +81 3 5841 3066
E-mail: asyanagi@mail.ecc.u-tokyo.ac.jp

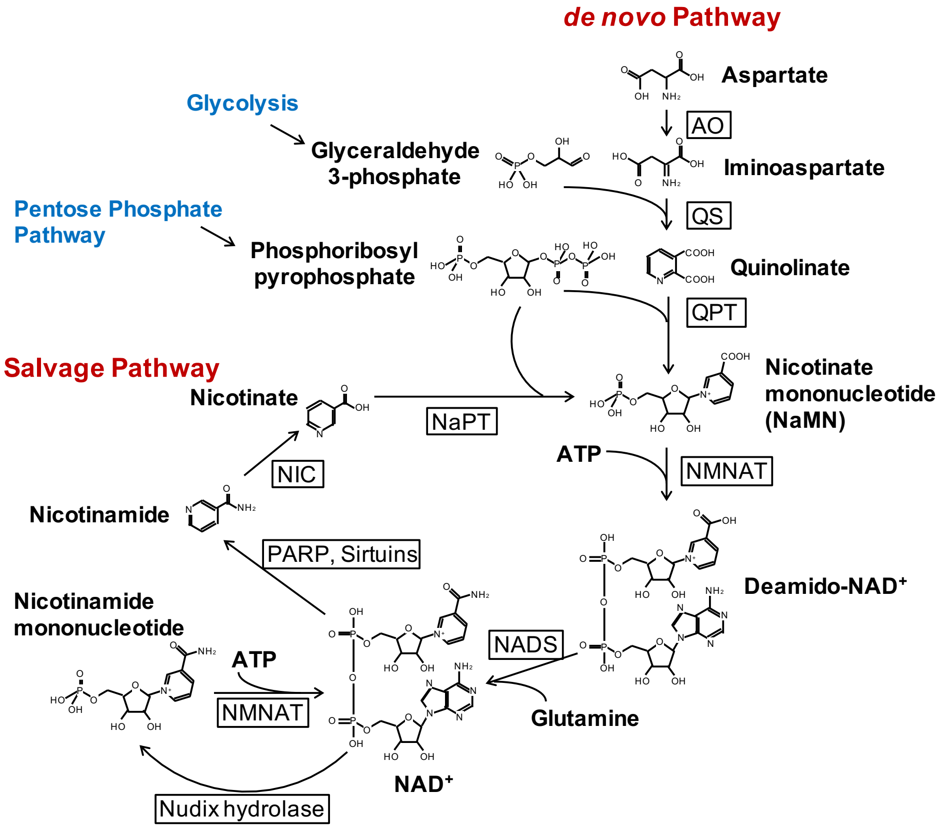

Supplementary Fig. 1 Metabolic pathway of NAD^+ biosynthesis in plants. AO, aspartate oxidase; QS, quinolinate synthase; QPT, quinolinate phosphoribosyltransferase; NMNAT, nicotinate mononucleotide adenyl transferase; NADS, NAD synthetase; PARP, poly(ADP-ribose) polymerase; NIC, nicotinamidase; NaPT, nicotinic acid phosphoribosyl transferase.

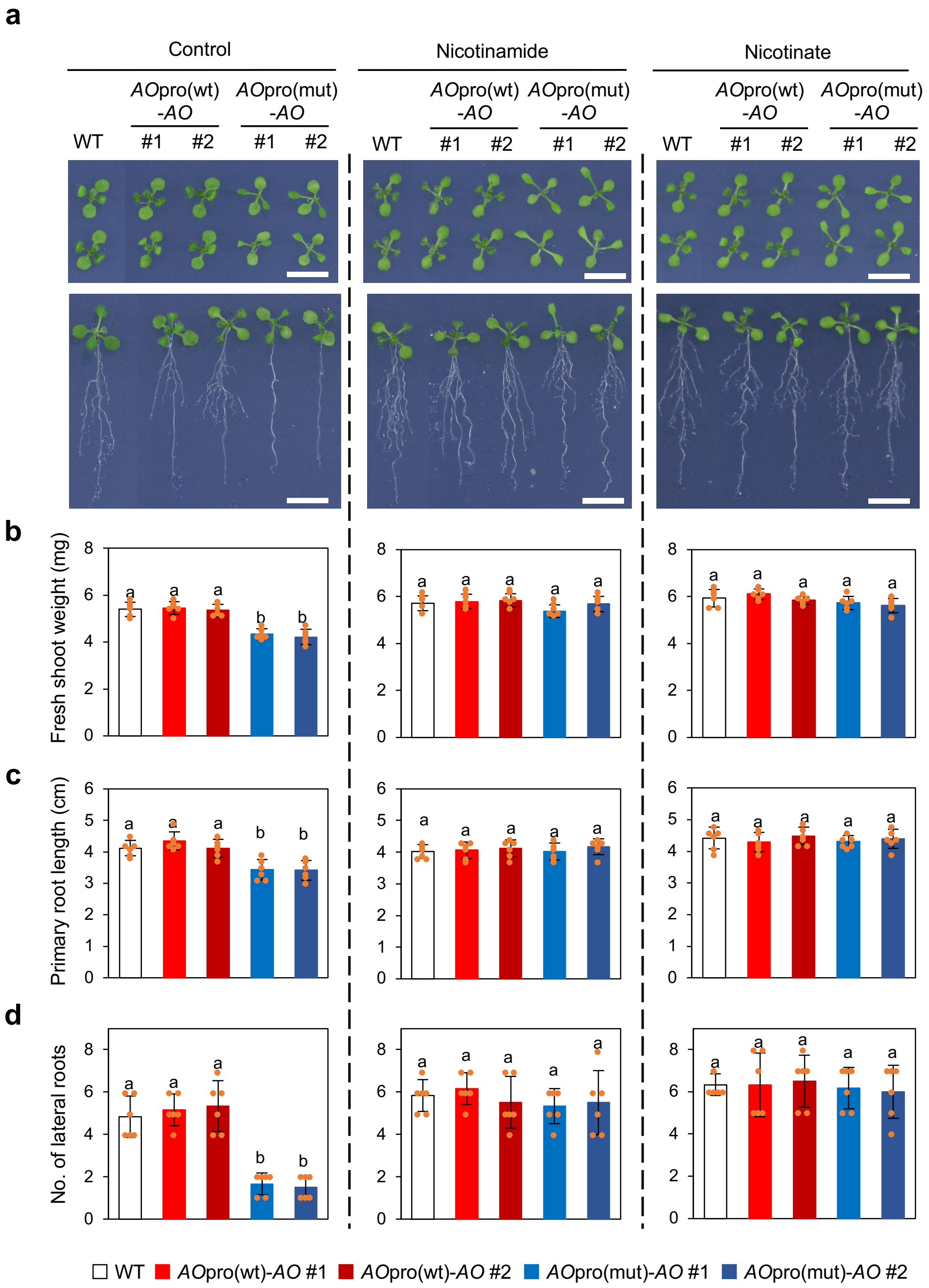

Supplementary Fig. 2 Recovery of growth defects and morphological abnormalities of *AO*pro(mut)-*AO* seedlings by the supplementation of nicotinamide or nicotinate. (a-d) Images of shoot and root growth (a), fresh shoot weight (b), primary root length (c), and the number of lateral roots (d) of WT, *AO*pro(wt)-*AO*, and *AO*pro(mut)-*AO* seedlings that were grown on 1/2MS agar plates for 4 d and then on agar plates containing 10 mM KNO_3 in the presence or absence (control) of 200 μM nicotinamide or 2 μM nicotinate for 6 d. Scale bar = 1 cm. In (b-d), data represent mean ± SD (n=6). Different letters denote statistically significant differences (Tukey's HSD test; $P < 0.05$).

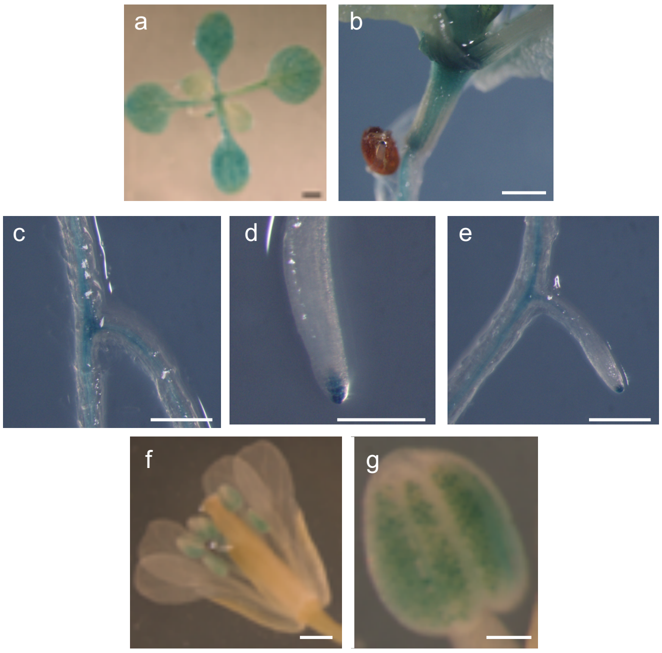

Supplementary Fig. 3 Histochemical GUS staining in transgenic plants expressing the *GUS* gene under the control of the *AO* promoter. Images showing the GUS staining of the shoot (a), shoot-root junction (b), main root-lateral root junction (c), primary root tip (d), and lateral root tip (e) of 10-d-old seedlings grown on 1/2MS agar plates, and of floral buds (c) and stamens (d) of 40-d-old plants grown in soil. Scale bars = 1 mm (a), 0.2 mm (b-e, g), and 0.5 mm (f).

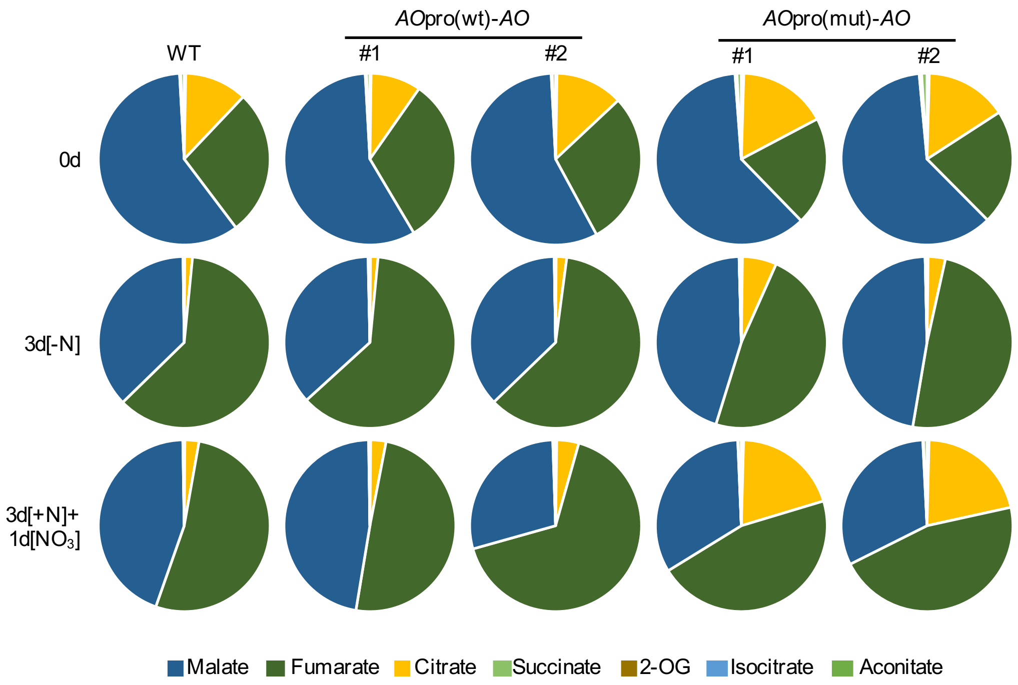

Supplementary Fig. 4 Pie charts illustrating the numerical proportion of metabolic intermediates in the TCA cycle in WT, *AO*pro(wt)-*AO*, and *AO*pro(mut)-*AO* plants under the indicated nutritional conditions. The contents of the intermediates in these plants under each nutrient condition are shown in Fig. 7.

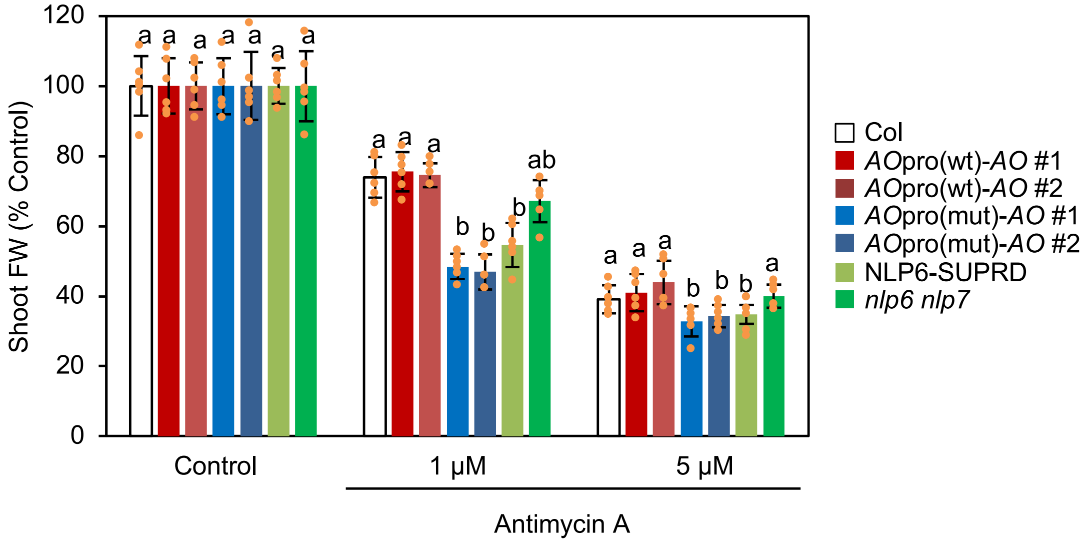

Supplementary Fig. 5 Effects of antimycin A on the fresh shoot weight. Seedlings were initially grown without antimycin A for 4 d and then in the presence of the indicated concentrations of antimycin A for 6 d (n = 6). WT plants, two independent lines (#1 and #2) of *AO*pro(wt)-*AO* and *AO*pro(mut)-*AO* plants, the NLP6-SUPRD transgenic line, and the *nlp6 nlp7* double mutant were used for the experiment. Different letters denote statistically significant differences (Tukey's HSD test; $P < 0.05$).

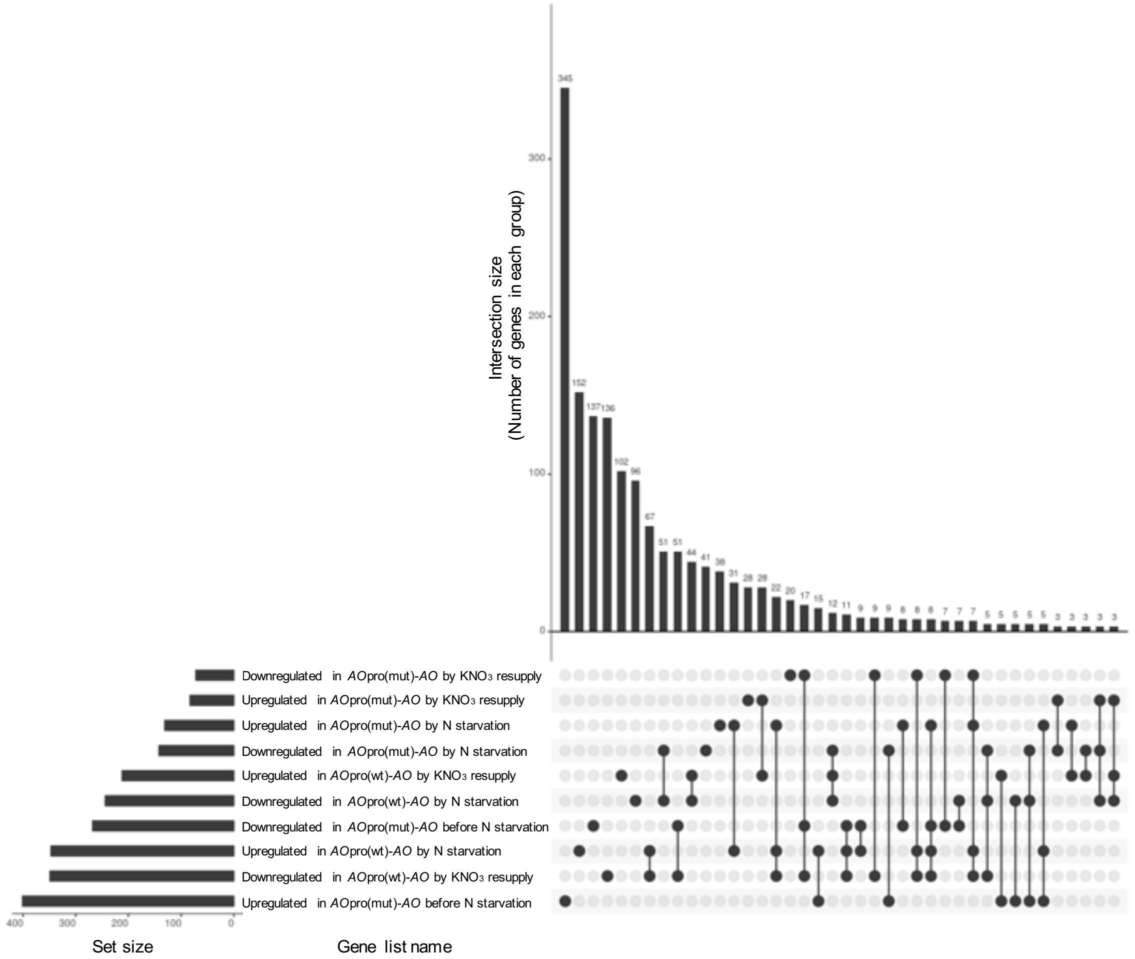

Supplementary Fig. 6 Overlaps among the 8 lists of genes upregulated or downregulated before N starvation or by N starvation or KNO3 resupply in *AO*pro(wt)-*AO* and *AO*pro(mut)-*AO* plants. The left bars (set size) indicate the number of genes in each gene list. Genes in the 8 gene lists are classified into 40 groups indicated by vertical bars (intersection size), depending on which gene lists include them. The length of vertical bars presents the number of genes included in each group. Black circles present groups consisting of a gene list, and therefore multiple black circles in a row indicate groups shared by multiple gene lists.
