# Supplementary material for: Arabidopsis nitrate-induced aspartate oxidase gene expression is necessary to maintain metabolic balance under nitrogen nutrient fluctuation
Source: Commun Biol. 2022 May 9;5:432. doi: 10.1038/s42003-022-03399-5 (PMC9085827; doi:10.1038/s42003-022-03399-5)
Supplement: Supplementary file 2 — Description of Additional Supplementary Files [file 42003_2022_3399_MOESM2_ESM.pdf]

Description of Additional Supplementary Files

File name: Supplementary Data 1
Description: Metabolite contents in the wildtype Arabidopsis plants and two independent lines of AOpro(wt)-AO and AOpro(mut)-AO plants.

File name: Supplementary Data 2
Description: Genes expressed to higher levels in AOpro(mut)-AO plants than in AOpro(wt)-AO plants when grown in 1/10MS medium for 20 d.

File name: Supplementary Data 3
Description: Genes expressed lower levels in AOpro(mut)-AO plants than in AOpro(wt)-AO plants when grown in 1/10MS medium for 20 d.

File name: Supplementary Data 4
Description: Gene ontology term enrichment analysis of differentially expressed by the loss of nitrate-induced AO expression at steady state.

File name: Supplementary Data 5
Description: Genes repressed by N starvation treatment in AOpro(wt)-AO plants.

File name: Supplementary Data 6
Description: Genes activated by N starvation treatment in AOpro(wt)-AO plants.

File name: Supplementary Data 7
Description: Genes activated by N starvation treatment in AOpro(mut)-AO plants.

File name: Supplementary Data 8
Description: Genes repressed by N starvation treatment in AOpro(mut)-AO plants.

File name: Supplementary Data 9
Description: Genes activated by KNO3 supply post-N starvation in AOpro(wt)-AO plants.

File name: Supplementary Data 10
Description: Genes repressed by KNO3 supply post-N starvation in AOpro(wt)-AO plants.

File name: Supplementary Data 11
Description: Genes activated by KNO3 supply post-N starvation in AOpro(mut)-AO plants.

File name: Supplementary Data 12
Description: Genes repressed by KNO3 supply post-N starvation in AOpro(mut)-AO plants.

File name: Supplementary Data 13
Description: Gene ontology term enrichment analysis of genes which responded to nitrogen starvation in AOpro(wt)-AO plants.

File name: Supplementary Data 14
Description: Gene ontology term enrichment analysis of genes which responded to nitrogen starvation in AOpro(mut)-AO plants.

File name: Supplementary Data 15
Description: Gene ontology term enrichment analysis of genes which responded to nitrogen re-supplement in AOpro(wt)-AO plants.

File name: Supplementary Data 16
Description: Gene ontology term enrichment analysis of genes which responded to nitrogen re-supplement in AOpro(mut)-AO plants.

File name: Supplementary Data 17
Description: PCR primers used for qPCR, cloning, genotyping, or preparation of DNA probes.
